# Weekly Dose-Dense Paclitaxel and Triweekly Low-Dose Cisplatin: A Well-Tolerated and Effective Chemotherapeutic Regimen for First-Line Treatment of Advanced Ovarian, Fallopian Tube, and Primary Peritoneal Cancer

**DOI:** 10.3390/ijerph16234794

**Published:** 2019-11-29

**Authors:** Min Cheng, Howard Hao Lee, Wen-Hsun Chang, Na-Rong Lee, Hsin-Yi Huang, Yi-Jen Chen, Huann-Cheng Horng, Wen-Ling Lee, Peng-Hui Wang

**Affiliations:** 1 Department of Obstetrics and Gynecology, Taipei Veterans General Hospital, Taipei 112, Taiwan; alchemist791025@gmail.com (M.C.); l.harvee@gmail.com (H.H.L.); whchang@vghtpe.gov.tw (W.-H.C.); nllee@vghtpe.gov.tw (N.-R.L.); chenyj@vghtpe.gov.tw (Y.-J.C.); hchorng@vghtpe.gov.tw (H.-C.H.); 2 Department of Obstetrics and Gynecology, National Yang-Ming University, Taipei 112, Taiwan; 3 Department of Nursing, Taipei Veterans General Hospital, Taipei 112, Taiwan; 4 Biostatics Task Force, Taipei Veterans General Hospital, Taipei 112, Taiwan; sweethsin509@gmail.com; 5 Institute of Clinical Medicine, National Yang-Ming University, Taipei 112, Taiwan; 6 Department of Medicine, Cheng-Hsin General Hospital, Taipei 112, Taiwan; 7 Department of Nursing, Oriental Institute of Technology, New Taipei City 220, Taiwan; 8 Department of Medical Research, China Medical University Hospital, Taichung 440, Taiwan; 9 The Female Cancer Foundation, Taipei 104, Taiwan

**Keywords:** dose-dense weekly paclitaxel, epithelial ovarian cancer, fallopian tube cancer, FIGO stage IIIC–IV, low-dose triweekly cisplatin, primary peritoneal serous carcinoma

## Abstract

A combination of cytoreductive surgery, either primary (PCS) or interval (ICS), and chemotherapy with a platinum-paclitaxel regimen is the well-accepted treatment for advanced-stage epithelial ovarian cancer (EOC), fallopian tube cancer (FTC), and primary peritoneal serous carcinoma (PPSC), but it is still uncertain whether a combination of dose-dense weekly paclitaxel and low-dose triweekly cisplatin is useful in the management of these patients. Therefore, we retrospectively evaluated the outcomes of women with advanced-stage EOC, FTC, and PPSC treated with PCS and subsequent dose-dense weekly paclitaxel (80 mg/m^2^) and low-dose triweekly cisplatin (20 mg/m^2^). Between January 2011 and December 2017, 32 women with International Federation of Gynecology and Obstetrics (FIGO) stage IIIC–IV EOC, FTC, or PPSC were enrolled. Optimal PCS was achieved in 63.5% of patients. The mean and median progression-free survival was 36.5 and 27.0 months, respectively (95% confidence interval (CI): 26.8–46.2 and 11.3–42.7 months, respectively). The mean overall survival was 56.0 months (95% CI: 43.9–68.1 months), and the median overall survival could not be obtained. The most common all-grade adverse events (AEs) were anemia (96.9%), neutropenia (50%), peripheral neuropathy (28.1%), nausea and vomiting (34.4%), and thrombocytopenia (15.6%). These AEs were predominantly grade 1/2, and only a few patients were complicated by grade 3/4 neutropenia (21.9%) and anemia (6.3%). A multivariate analysis indicated that only suboptimal PCS was significantly correlated with a worse prognosis, resulting in an 11.6-fold increase in the odds of disease progression. In conclusion, our data suggest that dose-dense weekly paclitaxel (80 mg/m^2^) combined with low-dose triweekly cisplatin (20 mg/m^2^) is a potentially effective and highly tolerable front-line treatment in advanced EOC, FTC, and PPSC. Randomized trials comparing the outcome of this regimen to other standard therapies for FIGO stage IIIC–IV EOC, FTC, and PPSC are warranted.

## 1. Introduction

Over the past decade, the predominant treatment for advanced epithelial ovarian cancer, fallopian tube cancer, and primary peritoneal serous cancer (EOC, FTC, and PPSC, respectively) has been primary cytoreductive surgery (PCS) plus adjuvant triweekly paclitaxel and carboplatin. This treatment strategy was based on a study by McGuire et al. [1] that demonstrated the superiority of incorporating paclitaxel into cisplatin-based regimens compared to cyclophosphamide plus cisplatin in patient survival. Further studies have confirmed significant survival benefits in women with EOC, FTC, and PPSC treated with a combination of triweekly cisplatin and paclitaxel in place of the original cisplatin–cyclophosphamide regimen [2,3]. Due to potential neural and renal toxicity as well as the high emetic effects of cisplatin, carboplatin has replaced cisplatin in this combination and has become a standard postoperative adjuvant therapy in the management of women with EOC, FTC, and PPSC after PCS [4,5,6,7,8,9,10,11,12]. In patients not suitable for PCS that require interval cytoreductive surgery (ICS), this triweekly carboplatin and paclitaxel regimen is also used as a neoadjuvant chemotherapy (NACT) for advanced-stage EOC, FTC, and PPSC patients [13,14,15,16,17,18,19]. Under this standard therapy, the median progression-free survival (PFS) is considered to be only between 16 and 21 months, and the median overall survival (OS) is between 32 and 57 months [1,3,4,5,6,7,8,9,12]. Therefore, many efforts have been made to enhance therapeutic effects and subsequently increase PFS and OS. These new modalities of treatment include altered delivery methods of antineoplastic drugs (intravenous or intraperitoneal routes), hyperthermia therapy, and the application of new agents, such as antiangiogenic drugs, immune checkpoint inhibitors, immune system modulators, and targeted therapy, including poly(adenosine diphosphate (ADP)-ribose) polymerase (PARP) inhibitors [11,12,15,20,21,22,23,24,25,26,27,28,29,30,31,32,33,34,35,36]. However, the high cost of all these treatment types is a cause for concern, which is further compounded by the need for long-term maintenance therapy in some agents.

The dose-dense regimen of paclitaxel stems from the Norton–Simon hypothesis, with the rationale that smaller tumors are more prone to eradication and that the chance of tumor regrowth could be decreased by administering an agent more frequently (metronomic therapy) [37,38]. An initial study of the dose-dense administration of weekly paclitaxel with triweekly carboplatin in Japanese Gynecologic Oncology Group (JGOG) 3016 showed significant improvements in median PFS and median OS for stage II to IV EOC [39], resulting in subsequent phase III trials of dose-dense paclitaxel versus triweekly paclitaxel combined with carboplatin in EOC treatment in predominantly western populations [40,41,42]. In addition to the Japanese trial, a study by Chan et al. revealed evidence of superior PFS with weekly paclitaxel and triweekly carboplatin compared to the standard regimen in patients who did not receive concurrent bevacizumab treatment [41]. Moreover, many studies using weekly paclitaxel in the management of patients with EOC have not limited its use to adjuvant therapy after PCS but have also found it acceptable in NACT [43,44,45,46,47,48]. However, the survival benefits found in dose-dense treatments have not always been reproducible in these studies [40,42].

Carboplatin is more myelosuppressive than cisplatin but has less gastrointestinal, renal, and neurologic toxicity, which is the main reason that it has replaced cisplatin in the platinum–paclitaxel regimen [6,7,8]. In this study, we would like to reconsider the role of cisplatin in the management of women with advanced-stage EOC, FTC, and PPSC, because cisplatin toxicity can be reduced through a reduction of the dosage. This regimen of dose-dense weekly paclitaxel plus low-dose triweekly cisplatin can be considered a modified form of previous dose-dense regimens in JGOG 3016 [39]. Our regimen uses low-dose cisplatin rather than carboplatin, with the expectation that this reduces myelosuppression and improves treatment tolerability while not compromising the therapeutic effects. This study aimed to explore the efficacy and safety of this new combination regimen of dose-dense weekly paclitaxel plus low-dose triweekly cisplatin for advanced EOC, FTC, and PPSC in an Asian population (a Chinese population).

## 2. Materials and Methods

### 2.1. Patient Population

This was a single-arm, single-institution retrospective cohort study. The eligible inclusion criteria were patients with International Federation of Gynecology and Obstetrics (FIGO) stage IIIC–IV histologically confirmed ovarian, primary peritoneal, or fallopian tube cancer who underwent PCS following a total of six cycles of dose-dense chemotherapy (weekly paclitaxel and triweekly cisplatin). Patients were excluded if they had NACT; had other newly diagnosed cancer, previous chemotherapy, or radiotherapy in the past two years; had a total relative dose intensity (RDI) less than 70% of standard doses for either paclitaxel or cisplatin after six cycles of treatment; or had simultaneous use of other antineoplastic agents, antiangiogenic agents, or targeted therapy. Informed consent was obtained from all eligible participants. This study was approved by the institutional review board.

### 2.2. Treatment

All patients received a dose-dense regimen of weekly paclitaxel and triweekly cisplatin. Paclitaxel 80 mg/m^2^ was administered over 2 h intravenously on days 1, 8, and 15, followed by intravenous infusion of cisplatin 20 mg/m^2^ for 1 h on day 1. Standard premedication with dexamethasone (20 mg), 2000 ml of normal saline, and palonosetron (250 *u*g) was prescribed intravenously to all patients on treatment day. Granulocyte colony-stimulating factor (GCSF) was administered to patients with grade 3/4 neutropenia for three days before chemotherapy. Treatment was delayed for 7 days in patients with febrile neutropenia to allow for antibiotics administration. Paclitaxel was reduced by 20% and cisplatin was withheld if febrile neutropenia or grade 3/4 neutropenia was noted. The estimated glomerular filtration rate (eGFR) was calculated according to the Cockcroft–Gault formula [49,50,51], and cisplatin was reduced by 50% if the eGFR decreased to 45–60 ml/min or was held for one cycle if the eGFR was less than 45 ml/min. 

### 2.3. Assessments

The first cycle of chemotherapy was administered after PCS within one week. Adverse effects (AEs) were evaluated before every cycle of treatment according to the National Cancer Institute’s Common Terminology Criteria for Adverse Events (NCI-CTCAE), version 5.0 [52,53]. Objective follow-up of disease status was assessed with computed tomography (CT) or magnetic resonance imaging (MRI), combined with clinical and CA-125 (cancer antigen 125, carcinoma antigen 125, or carbohydrate antigen 125) examinations [54,55,56]. An imaging evaluation for the response to treatment was performed according to the Response Evaluation Criteria in Solid Tumors (RECIST), version 1.1 [54,55,56]. Raised CA-125 levels alone did not indicate disease progression if measurable disease was not available in radiological or clinical examinations. For patients who had ever had normalized CA-125 levels after treatment, the CA-125 criteria for defining disease progression were raised values greater than two times the upper normal limit. For patients who did not have normalized CA-125 during treatment, disease progression was defined as a CA-125 value greater than 2 times the nadir value. Response to treatment was evaluated upon the completion of six cycles of chemotherapy and was reassessed every six months during the first two years and then every year thereafter. Additional imaging and CA-125 evaluation could be performed if there were clinical signs of suspected progressive disease (PD). 

### 2.4. Statistical Analysis

The primary endpoint was PFS, which was defined as the time from the date of primary operation to the earliest date of disease progression, death from any cause, or the date of the last known follow-up. The secondary endpoints were OS, the overall response rate (ORR), the clinical benefit rate (CBR), and safety. OS was defined as the time from the date of primary operation to the date of death from any cause or the date of the last known follow-up. Patients receiving optimal PCS without clinically or instrumentally measurable disease before the first cycle of treatment were not evaluated for response. PFS and OS were calculated using the Kaplan–Meier method. Risk factors for disease progression were evaluated with a logistic multivariate regression model. All statistical analyses were conducted using SPSS v. 24.0 (IBM Corp., Armonk, NY, USA).

## 3. Results

### 3.1. Clinical Characteristics and Pathological Status

Between January 2011 and December 2017, 32 eligible patients were identified through our electronic prescribing system. Table 1 summarizes the characteristics of all patients. The median age was 57 years (range: 33–79 years). All patients were primarily diagnosed with EOC, FTC, or PPSC that was stage IIIC (81.3%) or stage IV (18.8%) and that originated from bilateral ovaries (81.3%), fallopian tubes (6.3%), or the peritoneum (12.5%). The most prevalent histological type was high-grade serous carcinoma (56.3%). All patients received PCS, and the optimal debulking rate was 62.5%. Eighteen patients (56.3%) completed the six cycles of weekly paclitaxel and triweekly cisplatin without any delay or reduction of dosage for both antineoplastic agents (cisplatin and paclitaxel). Eleven patients (34.4%) had treatment delays of no more than one week, and only three patients (9.4%) had a delay time of more than two weeks.

### 3.2. Outcomes

**Figure 1:**
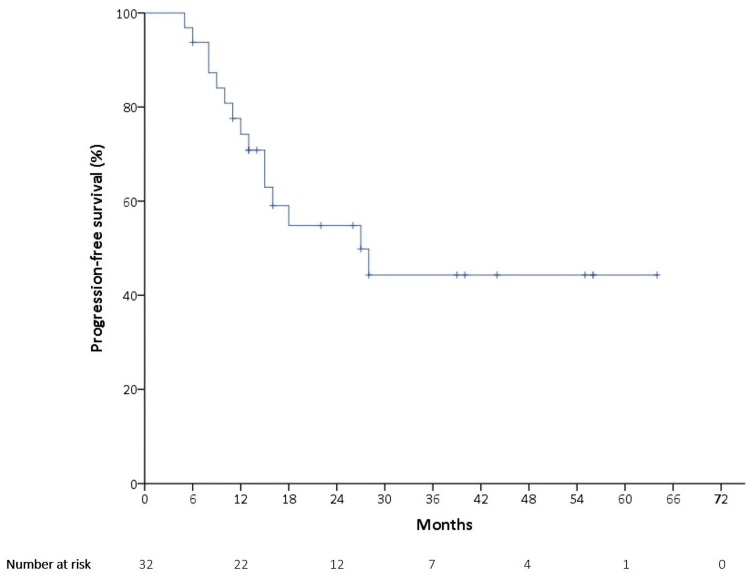
Kaplan–Meier estimates of progression-free survival (PFS).

**Figure 2:**
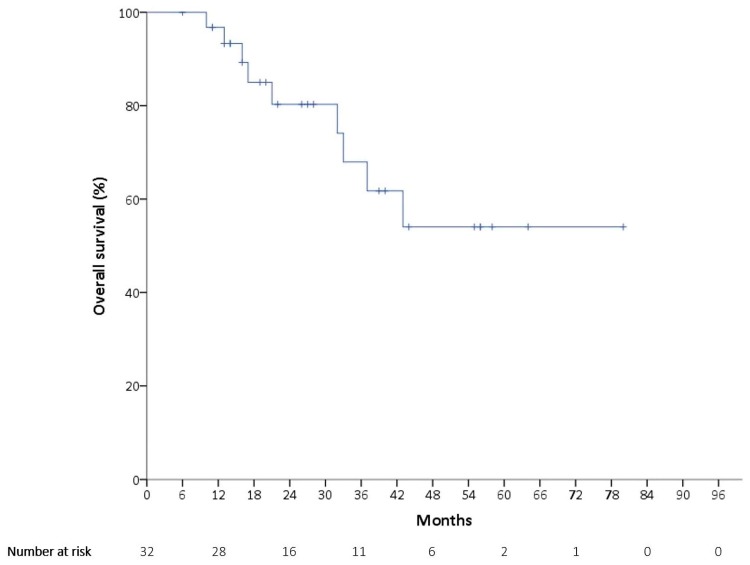
Kaplan–Meier estimates of overall survival (OS).

At the time of the data cutoff on 31 January 2019, the median follow-up time was 24 months, with disease progression occurring in 15 patients (46.9%). As is presented in Figure 1, the median PFS was 27 months (95% confidence interval (CI): 11.3–42.7 months). The mean PFS was 36.5 months, with a 95% CI of 26.8–46.2 months. There were a total of nine deaths (28.1%). The mean OS was 56.0 months (95% CI: 43.9–68.1 months), as is shown in Figure 2, and the median OS could not be obtained.

Objective response was evaluated by RECIST in the 12 patients who received suboptimal cytoreductive surgery with measurable disease at baseline. Two complete responses (16.7%) were observed, and both responding patients had a 4.5-month duration of response. Two patients had stable disease (16.7%), and the CBR was 33.3%.

### 3.3. Prognostic Factors

To clarify the prognostic factors for disease progression, a univariate analysis of clinicopathologic factors showed that endometrioid histology tended to be a relatively poor prognosis predictor (Table 2). However, it was not statistically significant in the multivariate analysis. After adjusting for histology, residual tumor size, and time of treatment, a multivariate analysis indicated a significantly worse prognosis in residual tumor sizes greater than 1 cm, with an 11.6-fold increase in the odds of disease progression. This in turn also implied a better prognosis in patients receiving optimal cytoreductive surgery. To investigate the possible interactions between variables, a variance inflation factor (VIF) was used for assessing multicollinearity. All variables displayed VIF values less than 5, which ensured the absence of collinearity in the multivariate regression analysis.

### 3.4. Adverse Events

Table 3 lists the adverse events, and no treatment-related death was observed. The most common all-grade AEs were anemia (96.9%), neutropenia (50%), and nausea and vomiting (34.4%). However, only 21.9% and 6.3% of patients had grade 3/4 neutropenia and anemia, respectively. There was no grade 3/4 thrombocytopenia, and only five patients (15.6%) had grade 1/2 thrombocytopenia. Of note, there was no grade 3/4 kidney injury, proteinuria, sensory neuropathy, nausea, or vomiting given the propensity for these cisplatin toxicities. There were no patients withheld from cisplatin administration due to impaired renal function.

## 4. Discussion

The modification of dose scheduling and intensity is one of the targeted strategies for improving the prognosis of advanced EOC, FTC, and PPSC [39,40,43,50]. Our study tried to evaluate the outcome of patients with FIGO stage IIIC–IV EOC, FTC, and PPSC treated with dose-dense weekly paclitaxel and low-dose triweekly cisplatin regimen. The primary outcome of the current study was PFS, and the results seem to be promising because the median PFS (27 months) was longer than in previous western trials regarding dose-dense chemotherapy for advanced EOC, FTC, and PPSC (median PFS: 14.2–24.9 months) [41,42]. Moreover, it was even longer than in results from the experimental and control arms of many studies [57] that have attempted to add another agent to standard chemotherapy, regardless of whether the agents were given simultaneously during front-line chemotherapy or during maintenance therapy after standard chemotherapy [10,11,12,57,58,59,60,61,62,63,64,65]. These adding agents have included antiangiogenic drugs, PARP inhibitors, immune system modulators, and many multitarget compounds that were used as upfront therapy [10,11,12,23,27,28,29,30,31,32,57,58,59,60,61,62,63,64,65]. 

The first two positive advanced-stage frontline ovarian cancer randomized phase III trials that added bevacizumab to chemotherapy were Gynecologic Oncology Group study 0218 (GOG-0218) and Gynecologic Cancer InterGroup (GCIG) International Collaboration on Ovarian Neoplasms (ICON7) [57,58,59,60,61]. These two trials used different treatment durations and dosages of bevacizumab (a dose of 15 mg/kg for 22 cycles in GOG-0218 and a dose of 7.5 mg/kg for 18 cycles in ICON7), but they both showed an increase in PFS [57]. In GOG-0218, the median PFS was 14.1 months in the bevacizumab-concurrent plus maintenance arm compared to 10.3 months in the standard chemotherapy arm, with a statistically significant increase of 4 months [10,57,59]. A similar positive finding of prolonged PFS in patients treated with bevacizumab-concurrent plus maintenance therapy was noted in ICON7, with an increase of 1.5 months (from 20.3 months to 21.8 months) compared to standard chemotherapy alone [57,60,61]. The ICON7 study further identified the apparent benefits of adding bevacizumab in selective highly risky patients, such as patients with FIGO IIIC and FIGO V, who could not reach initially optimal PCS, where the estimated median PFS was 10.5 months in the standard chemotherapy arm compared to 15.9 months in the bevacizumab-concurrent plus maintenance arm [57]. Our results seemed to be not inferior to the results from patients treated with standard therapy plus bevacizumab treatment, as shown above [10,57,58,59,60,61], and also not inferior to the data from the Japanese trial (median PFS: 28.2 months) [39].

One multitargeted compound, nintedanib (an oral triple angiokinase inhibitor of the vascular endothelial growth factor receptor (VEGFR), platelet-derived growth factor receptor (PDGFR), and fibroblast growth factor receptor (FGFR)), has been used for maintenance therapy in patients with advanced-stage EOC who were given standard-of-care PCS and carboplatin plus paclitaxel chemotherapy, and the results showed that the median PFS was significantly longer in the nintedanib group than in the placebo group (17.2 months vs 16.6 months) [62]. The maintenance of pazopanib, another oral multikinase inhibitor of VEGFR -1/-2/-3, PDGFR -α/-β, and c-Kit, also prolonged PFS compared to a placebo (a median of 17.9 months vs 12.3 months) in patients with advanced EOC who had not progressed after first-line standard chemotherapy [63]. The median PFS in our current study seemed to be not inferior to the patients treated with maintenance therapy, as is shown above. 

The input of immune system modulators in ovarian cancer is based on the observation that immunosuppressive microenvironments can affect tumor growth, metastasis, and even treatment resistance [64]. Therefore, additional therapies might be needed. There are many new cancer-targeted strategies available in the management of patients with advanced-stage EOC, and some of them are new combinations [57,64,65]. For example, several phase III trials, including NRG-GY009 (NCT02839707), ATLANTE (NCT02891824), IMagyn050 (NCT03038100), NRG-GY005 (NCT02502266) phase II/III, and NRG-GY004 (NCT02446600), are ongoing, and many are combinations of multiagents during or after standard-of-care PCS/ICS and carboplatin plus paclitaxel chemotherapy (adjuvant chemotherapy or NACT) [57,64]. 

The biggest change in EOC treatment might have been explored by the SOLO-1 study, which demonstrated that the risk of disease progression was 70% lower with olaparib (estimated median PFS ≥49 months) than with a placebo (median PFS of 13 months) in patients with newly diagnosed advanced ovarian cancer who had a complete or partial response to platinum-based chemotherapy [25]. However, the benefits might be limited to certain populations, such as patients with a mutation of breast cancer gene 1 (BRCA1), breast cancer gene 2 (BRCA2), or both (BRCA1/2). 

Studies by Katsumata et al. [39], Chan et al. [41], and Walker et al. [42] have shown an optimal debulking rate ranging from 37% to 92%. The distinctly lower PFS in the study by Chan et al. could be explained by the lower optimal debulking rate and the more advanced-stage disease [41]. In our study, the optimal debulking rate was 63.5%, which was comparable to previous trials [39,42]. 

In the subgroup of patients who received suboptimal PCS, the change in tumor size could be used to evaluate the treatment response, since residual tumors over 1 cm can be detected by CT or MRI. As expected, the ORR of the suboptimal debulking group was low, which could be explained by the highly resistant nature of grossly larger tumors under the Norton–Simon hypothesis [37]: through the multivariate analysis in our study, we reaffirmed the necessity of achieving optimal PCS to significantly reduce the disease progression rate. Despite the poor prognosis for advanced disease with grossly residual tumors, the CBR could reach as high as 33.3% following dose-dense paclitaxel with low-dose cisplatin chemotherapy. Patients receiving NACT were not eligible in the current study to avoid possible interference with the follow-up evaluation of treatment effects. In contrast, NACT therapy has been included in previous studies of dose-dense chemotherapy regimens [39,41,42,48]. 

The current low-dose cisplatin plus dose-dense paclitaxel regimen was associated with lower rates of hematologic toxicities compared to the conventional dose-dense paclitaxel plus carboplatin regimen. The most prevalent AE in the current study was anemia of any grade (96.9%), and the second most prevalent was neutropenia of any grade (50%). However, in terms of grade 3 and grade 4 neutropenia, anemia, and thrombocytopenia, the low-dose cisplatin regimen had much fewer AEs than the standard-dose carboplatin regimen did, with the former only causing 21.9% of patients to have grade 3/4 neutropenia and 6.3% to have grade 3/4 anemia: there was an absence of grade 3/4 thrombocytopenia. Conventional dose-dense paclitaxel plus carboplatin studies have displayed grade 3/4 neutropenia, grade 3/4 anemia, and grade 3/4 thrombocytopenia in the range of 72–92%, 27–69%, and 18–44% (Table 4), respectively. The variation could have resulted from different carboplatin doses equivalent to the area under the curve (AUC), with both 5 and 6 used [39,41,42]. Concerning specific AEs associated with cisplatin, our study showed a low frequency of gastrointestinal, renal, and peripheral neuropathies of any grade, as well as an absence of grade 3 and grade 4 events, which were relatively lower than the reported grade 3 and grade 4 data in studies with carboplatin regimens [38,41,42]. These findings are noteworthy, since compared to conventional dose-dense paclitaxel plus carboplatin regimens, this current low-dose cisplatin plus dose-dense paclitaxel regimen had significantly lower rates of severe hematologic toxicities, and cisplatin-specific AEs were not evident with this relatively lower dose of cisplatin.

The limitations of this study included its retrospective design and small sample size, since we were practicing a relatively new regimen of chemotherapy in the primary treatment of advanced EOC, FTC, and PPSC. In addition, we did not evaluate new therapeutic strategies, such as maintenance therapy, in the current report. The nature of a single-arm study design did not allow us to compare treatment effects between our newly proposed therapy and current standard therapies. 

## 5. Conclusions

In conclusion, our data suggest that weekly dose-dense paclitaxel combined with triweekly low-dose cisplatin is a potentially effective and highly tolerable front-line treatment in advanced EOC, FTC, and PPSC. Randomized trials comparing the therapeutic outcomes of this regimen to other standard therapies for FIGO stage IIIC–IV EOC, FTC, and PPSC patients are warranted.

## Figures and Tables

**Table 1:** Demographic and clinicopathological characteristics.

Characteristics	Total (*n* = 32)
Age at diagnosis (years)	57 (33–79)
Age > 60 years	11 (34.4%)
FIGO stage	
IIIC	26 (81.3%)
IV	6 (18.8%)
Cancer type	
Ovarian	26 (81.3%)
Peritoneum	4 (12.5%)
Fallopian tube	2 (6.3%)
Histology	
High-grade serous	18 (56.3%)
Mucinous	1 (3.1%)
Clear cell	3 (9.4%)
Endometrioid	8 (25%)
Others (mixed high-grade serous)	2 (6.3%)
Size of residual tumor	
≤1 cm	20 (62.5%)
>1 cm	12 (37.5%)
Site of residual tumor	
Lower abdomen	12 (37.5%)
Upper abdomen	11 (34.4%)
Whole abdomen	9 (28.1%)
Time of treatment	
18 weeks	18 (56.3%)
18–21 weeks	11 (34.4%)
21–24 weeks	0 (0)
>24 weeks	3 (9.4%)
ECOG	
0–1	30 (93.8%)
2–3	2 (6.3%)

FIGO: International Federation of Gynecology and Obstetrics; ECOG: Eastern Cooperative Oncology Group Performance Status; Data are presented as a number (%) or the median (range).

**Table 2:** Association between baseline characteristics and the progression of disease.

Characteristic	Number (%)	Univariate Analysis		Multivariate Analysis	
		Odds Ratio (95% Confidence Interval (Cl))	*p*-Value	Odds Ratio (95% Cl)	*p*-Value
Age					
≤60 years	21 (65.6)	Reference			
>60 years	11 (34.4)	1.6 (0.37–6.95)	0.53		
FIGO stage					
IIIC	26 (81.3)	Reference			
IV	6 (18.8)	0.5 (0.08–3.22)	0.466		
Histology					
Serous/others	20 (62.5)	Reference		Reference	
Mucinous/clear cell	4 (12.5)	0.67 (0.08–5.75)	0.712	0.41 (0.03–5.25)	0.494
Endometrioid	8 (25)	0.1 (0.01–0.93)	0.043	0.11 (0.01–1.35)	0.085
Size of residual tumor					
≤1 cm	20 (62.5)	Reference		Reference	
>1 cm	12 (37.5)	3.71 (0.82–16.84)	0.089	11.6 (1.07–125.92)	0.044
Site of residual tumor					
Lower abdomen	12 (37.5)	Reference			
Upper abdomen	11 (34.4)	1.67 (0.31–9.01)	0.553		
Whole abdomen	9 (28.1)	4 (0.64–25.02)	0.138		
Time of treatment					
18 weeks	18 (56.3)	Reference		Reference	
18–21 weeks	11 (34.4)	2.75 (0.58–12.98)	0.201	5.17 (0.63–42.45)	0.126
>24 weeks	3 (9.4)	0.79 (0.06–10.38)	0.855	0.10 (0.004–2.26)	0.146
ECOG					
0–1	30 (93.8)	Reference			
2–3	2 (6.3)	1.14 (0.07–20.02)	0.927		

Data are presented as numbers (%).

**Table 3:** Adverse events (*n* = 32).

Events	Any Grade, *n* (%)	Grade 1/2, *n* (%)	Grade 3/4, *n* (%)
Neutropenia	16 (50)	9 (28.1)	7 (21.9)
Anemia	31 (96.9)	29 (90.6)	2 (6.3)
Thrombocytopenia	5 (15.6)	5 (15.6)	0
Renal toxicity	3 (9.4%)	3 (9.4)	0
Proteinuria	6 (18.8)	6 (18.8)	0
Peripheral neuropathy	9 (28.1)	9 (28.1)	0
Nausea	11 (34.4)	11 (34.4)	0

*n*: Number of patients; data are presented as numbers and percentages.

**Table 4:** Summary of treatment efficacy and safety of weekly paclitaxel and triweekly carboplatin in phase III RCTs.

Authors	Population	*n*	Regimen (Intravenous)	Median PFS	Median OS	Wbc	Plt	Rbc	SN	V
Katsumata et al. [39]	EOC FIGO II–IV	312	P 80mg/m^2^ (D1,8,15), C AUC 6 (D1)	28.2 months	100.5 months	92%	44%	69%	7%	3%
Chan et al. [41]	EOC FIGO II–IV	340	P 80mg/m^2^ (D1,8,15), C AUC 6 (D1), optional bevacizumab 15mg/kg (D1)	14.7 months	-	72%	20%	36%	3%	6%
		55	P 80mg/m^2^ (D1,8,15), C AUC 6 (D1), without bevacizumab	14.2 months	-	-	-	-	-	-
Walker et al. [42]	EOC FIGO II–IV	521	P 80mg/m^2^ (D1,8,15), C AUC 6 (D1), bevacizumab 15mg/kg (D1)	24.9 months	75.5 months	72%	18%	27%	6%	5%

All studies permitted the inclusion of patients receiving neoadjuvant chemotherapy; RCT: randomized control trial; *n*: number of patients; PFS: progression-free survival; OS: overall survival; Wbc: neutropenia; Plt: thrombocytopenia; Rbc: anemia; SN: sensory neuropathy; V: vomiting; EOC: epithelial ovarian cancer; FIGO: International Federation of Gynecology and Obstetrics; P: paclitaxel; D: day; C: carboplatin; AUC: area under the curve; kg: kilograms. The current adverse events (neutropenia, thrombocytopenia, anemia, sensory neuropathy, and vomiting) are limited to grade 3 and grade 4.

## Abbreviations

AEs	Adverse events
AUC	Area under the curve
CBR	Clinical benefit rate
CT	Computed tomography
CI	Confidence interval
EOC	Epithelial ovarian cancer
eGFR	Estimated glomerular filtration rate
FTC	Fallopian tube cancer
FIGO	International Federation of Gynecology and Obstetrics
ISC	Interval cytoreductive surgery
NCI-CTCAE	National Cancer Institute’s Common Terminology Criteria for Adverse Events
NACT	Neoadjuvant chemotherapy
MRI	Magnetic resonance image
ORR	Overall response rate
OS	Overall survival
PARP inhibitors	Poly(ADP-ribose) polymerase (PARP) inhibitors
PPSC	Primary peritoneal serous carcinoma
PSC	Primary cytoreductive surgery
PFS	Progression-free survival
PD	Progressive disease
RDI	Relative dose intensity
RECIST	Response Evaluation Criteria in Solid Tumors
RR	Risk ratio
VIF	Variance inflation factor
